# Methyl Donor-Deficient Diet during Development Can Affect Fear and Anxiety in Adulthood in C57BL/6J Mice

**DOI:** 10.1371/journal.pone.0105750

**Published:** 2014-08-21

**Authors:** Daisuke Ishii, Daisuke Matsuzawa, Shingo Matsuda, Haruna Tomizawa, Chihiro Sutoh, Eiji Shimizu

**Affiliations:** 1 Department of Cognitive Behavioral Physiology, Chiba University Graduate School of Medicine, Chiba, Japan; 2 Research Center for Child Mental Development, Graduate School of Medicine, Chiba University, Chiba, Japan; 3 Department of Ultrastructural Research, National Institute of Neuroscience, National Center of Neurology and Psychiatry, Tokyo, Japan; University of Missouri, United States of America

## Abstract

DNA methylation is one of the essential factors in the control of gene expression. Folic acid, methionine and choline (methyl donors)–all nutrients related to one-carbon metabolism–are known as important mediators of DNA methylation. A previous study has shown that long-term administration of a diet lacking in methyl donors caused global DNA hypermethylation in the brain (Pogribny et al., 2008). However, no study has investigated the effects of a diet lacking in methyl donors during the developmental period on emotional behaviors such as fear and anxiety-like behavior in association with gene expressions in the brain. In addition, it has not been elucidated whether a diet supplemented with methyl donors later in life can reverse these changes. Therefore, we examined the effects of methyl donor deficiency during the developmental period on fear memory acquisition/extinction and anxiety-like behavior, and the relevant gene expressions in the hippocampus in juvenile (6-wk) and adult (12-wk) mice. We found that juvenile mice fed a methyl-donor-deficient diet had impaired fear memory acquisition along with decreases in the gene expressions of *Dnmt3a* and *Dnmt3b*. In addition, reduced anxiety-like behavior with decreased gene expressions of *Grin2b* and *Gabar2* was observed in both the methyl-donor-deficient group and the body-weight-matched food-restriction group. After being fed a diet supplemented with methyl donors ad libitum, adult mice reversed the alteration of gene expression of *Dnmt3a*, *Dnmt3b*, *Grin2b* and *Gabar2*, but anxiety-like behavior became elevated. In addition, impaired fear-memory formation was observed in the adult mice fed the methyl-donor-deficient diet during the developmental period. Our study suggested that developmental alterations in the one-carbon metabolic pathway in the brain could have effects on emotional behavior and memory formation that last into adulthood.

## Introduction

Many mental disorders including anxiety disorders such as specific phobia, panic disorder, post-traumatic disorder (PTSD) and mood disorders develop in early life stages [1], [2], [3]; the age of onset of anxiety disorders is earlier than those of other disorders [4]. Importantly, there are notable individual differences in vulnerability, resilience and treatment response. For example, the Tokyo subway sarin attack in 1995 by a radical cult produced severe PTSD in some bystanders, but not to others. Gray-matter volume reductions in the left anterior cingulate cortex were observed only in the victims who developed PTSD [5]. Standard treatments of medication or psychotherapy such as cognitive behavior therapy (CBT) are beneficial in some patients, but not in others [6]. Clearly, individual factors play roles in the pathogenesis, development, and treatment response of mental disorders.

Genetic factors are involved in such variations. Epigenetic regulation, for instance through DNA methylation and histone acetylation, is one of the essential factors in the control of gene expression [7], [8]. To date, it has been shown that DNA methylation and histone acetylation are dynamically regulated in the adult nervous system [9], [10], [11], [12]. In particular, several studies have shown that the alteration of DNA methylation is involved in the pathology of mental disorders such as depression and anxiety disorders, and cognitive functions such as memory formation [13], [14], [15], [16]. Many twin studies have revealed that the onsets of various diseases including psychiatric disorders are not the same in monozygotic twins [17], [18], [19], [20]. In addition, there is a report that although twins are epigenetically indistinguishable during the early years of life, older monozygous twins exhibit remarkable differences in their overall content and genomic distribution of 5-methylcytosine DNA and histone acetylation [21]. These studies suggest that the growth environment after birth affects development of various diseases through the mediation of epigenetic changes.

B-vitamin folate, methionine and choline are essential for one-carbon transfer reactions including DNA methylation, which regulates the transfer of methyl groups in biological methylation reactions; these nutrients work as methyl donors [22], [23]. Deprivation of methyl donors such as Vitamin B and choline during pregnancy in rats has been revealed to affect the pups’ internal organs such as the gastric mucosa [24]. Rat pups from dams fed diets lacking methyl donors showed long-lasting disabilities in exploratory behavior and learning and memory capacities [25], and reduction of the thickness of the CA1 pyramidal layer in the hippocampus along with spatial memory impairment [26]. Importantly, a previous study revealed that the long-term (18/36 weeks) reduction of dietary methyl donors after weaning induced alteration of epigenetic status in the rat brain [27], although its effect on behavior was not investigated. In addition, a previous study of the postmortem human hippocampus [28] showed that the methylation state was different in suicide victims with child abuse from those without child abuse; thus, epigenetic alteration in specific genes during early childhood potentially contributes to the later development of mental disorders.

Given this background, we aimed to investigate the potential effect of epigenetic rearrangement during the maturing brain on the future development of fear and anxiety. In this study, we examined the effects on mice of a diet lacking methyl donors–namely methionine, choline, and folic acid–during the developmental period after weaning (3 to 6 weeks old), on fear-memory formation and anxiety-like behavior in juvenile (6-week-old) mice using contextual fear conditioning and the elevated plus-maze. Then, by supplying a normal diet after 6 weeks, we examined whether the effects of methyl-donor deficiency during the early phase would last or recover in adult (12-week-old) mice. In addition, to investigate the background genetic contribution to the behavioral alterations observed in the methyl-donor-deficient diet, we assessed the mRNA expression levels of DNA methyltransferases (*Dnmt 1*, *3a* and *3b*), NMDA receptor subunits (NR1, NR2A and NR2B) and GABA receptor subunits (*Gaba a2* and *a3*).

## Materials and Methods

### Ethics Statement

All mice were treated and cared for in accordance with the guidelines of Chiba University. All mice were sacrificed by cervical dislocation. The protocol was approved by the Committee on the Ethics of Animal Experiments of Chiba University (approval number, 24–187).

### Animals

Two-week-old C57BL/6J male mice were purchased from Nihon SLC (Hamamatsu, Shizuoka, Japan) and housed with 8-week-old female mice for one week. At 3 weeks of age, the male mice were transferred to experimental cages (4–5 mice/cage) kept at a controlled temperature (23±1°C) and on a 12-h light/dark cycle (light on at 07∶00 hours). The mice were randomly assigned to three groups. From 3 to 12 weeks of age (the end of the experiment), the control group received ad libitum a diet with strictly defined amounts of L-amino acids (methyl donors): 1.7 g/kg L-methionine, 0.2 g/kg folate acid and 14.48 g/kg choline (Dyets, Bethlehem, PA). A diet lacking in L-methionine, folic acid and choline (Dyets, Bethlehem, PA) was fed ad libitum to an experimental, folate-methionine-choline deficient (FMCD) diet group, from 3 to 6 weeks of age. Because the feeding of a methionine-choline-deficient diet was reported to induce the loss of body weight [29], we set up a third group under food restriction (FR) conditions, which received only 25% the amount of regular chow as the control group from 3 to 6 weeks of age, so that their body weights would be balanced with the FMCD group. Following these protocols, from 6 to 12 weeks of age the FMCD group and FR groups were fed ad libitum the control diet (Dyets, Bethlehem, PA). Body weights and food consumption were recorded weekly. All behavioral testing was conducted between 09∶00 and 15∶00 hours. Mice were randomized and used only once for each experiment. The research and animal care were carried out according to the Guide for Animal Experimentation of the Chiba University Graduate School of Medicine.

### Behavioral experiments

To explore the effect of the FMCD diet on both fear memory formation and anxiety-related behaviors, we used the contextual fear conditioning/paradigm with memory test and the elevated plus maze test on mice at both 6 and 12 weeks of age.

### Contextual fear conditioning and memory test

Experiments were carried out in a 22.8×19.7×13 cm experimental footshock chamber, with transparent walls in the front and back, stainless-steel bars, and a metal-grid floor connected to a shock scrambler and generator in a sound-attenuating box. Habituation started 1 day before the contextual fear conditioning and consisted of two 20-min-long pre-exposure periods to the footshock chamber. The interval between the periods was 2 h, during which no shock was delivered. Twenty-four hours after habituation, we conducted contextual fear conditioning without any cues (tones or lights). After a 120-s acclimation period, all groups of mice received 3 footshocks (US; 2-s, 0.75 mA, 120-s inter-trial interval). The activity of the mice was monitored by FreezeFrame (Actimetrics Software, Wilmette, IL). Freezing (no visible movement except respiration) was measured using a digital video camera connected to a computer with Actimetrics FreezeFrame software (Actimetrics) every 5 s and converted to a percentage [(freezing observations/total observations)×100]. Twenty-four hours after contextual fear conditioning, a memory test was performed. The mice were placed for 3.5 min without footshocks in the same experimental chamber where the footshocks were delivered. The experimental footshock chamber was cleaned with 70% ethanol before and after use.

### Elevated plus-maze

The elevated plus-maze (EPM) was made of black Plexiglas and consisted of two open arms (30 cm×5 cm) and two closed arms (30 cm×5 cm×20 cm) extending from a central platform (5 cm×5 cm). The maze was elevated 50 cm above the floor. Each mouse was placed at the center of the maze facing an open arm and allowed to explore freely for 5 min. During the 5-min test period, (1) the total distance traveled, (2) the time spent in the open arms, and (3) the number of stretch-attend postures (risk assessments) were automatically recorded and analyzed by a video tracking software (TopScan, Primetech Corporation, Tokyo). The apparatus was thoroughly cleaned with water after removal of each mouse.

### Tissue preparation

Mice were sacrificed by cervical dislocation at 6 and 12 weeks of age. Upon sacrifice, the brains were quickly removed and immediately rinsed in ice-cold phosphate-buffered saline (PBS; pH 7.4). Slices 1-mm-thick were prepared using a mouse coronal brain matrix (RBM-2000C, ASI Instruments, Warren, MI), and the tissue was dissected out in ice-cold PBS under a stereoscopic microscope. The dorsal hippocampus was dissected out by a sharp scalpel. These tissues were placed in dry-ice-chilled 1.5-ml microcentrifuge tubes, immediately frozen in liquid nitrogen and stored at −80°C until further use.

### RNA extraction and quantitative real-time PCR

Total RNA was extracted from the dorsal hippocampus using the Qiagen Allprep RNA/DNA Mini Kit (QIAGEN, Alameda, CA) as instructed to purify total RNA. The concentration and integrity of RNA were measured with the NanoDrop 2000 spectrophotometer (Thermo Scientific Wilmington, DE). cDNA was synthesized using the High Capacity cDNA Reverse Transcription Kit (Applied Biosystems, Foster City, CA) according to the manufacturer’s instructions. Ninety ng of total RNA were reverse transcribed into cDNA in a 20-µl reaction volume using MultiScribe Reverse Transcriptase according to the manufacturer’s instructions. The program was as follows: 25°C for 10 min, 37°C for 120 min, and 85°C for 5 min. cDNA was stored at −20°C until further use. Real-time PCR was performed with the Applied Biosystems 7300 Real-Time PCR System (Applied Biosystems, Foster City, CA) in a total volume of 15 µl. Gene expressions were analyzed using FastStart TaqMan Probe Master (Rox) (Roche Molecular Biochemicals, Mannheim) containing 1.5 µl of cDNA, 10 µM of the probe (Universal Probelibrary probe, Roche) and 20 µM of both forward and reverse primers (Table 1). After incubation for 10 min at 95°C, the reaction continued for 40 cycles at 95°C for 15 s and 60°C for 1 min. Amplification data were analyzed by instrument software (SDS, Applied Biosystems). Standard curves for each gene expression were generated, each mRNA expression was calculated from the standard curve, and quantitative normalization of cDNA in each sample was performed using expression of the *Gapdh* gene as an internal control. The results of mRNA expression were presented as fold increases. Real-time PCR assays were tested in duplicate for each sample, and a mean value was used for calculation of expression levels.

**Table 1:** Primers for Real-time PCR.

Gene name	Forward primer	Reverse primer
*Dnmt1*	5′ - caaatagatccccaagatccag - 3′	5′ - cggaactaggtgaagtttcaaaaa - 3′
*Dnmt3a*	5′ - ctttgatgggattgctacagg - 3′	5′ - acacctcggaggcaatgtag - 3′
*Dnmt3b*	5′ - atgatcgatgccatcaaggt - 3′	5′ - gggaagccgaagatcctg - 3′
*Grin1*	5′ - tgtcatcccaaatgacagga - 3′	5′ - gggttcttggtggattgtca - 3′
*Grin2a*	5′ - attcaaccagaggggcgta - 3′	5′ - ttcaagacagctgcgtcatag - 3′
*Grin2b*	5′ - tcatggtatcacgcagcaat - 3′	5′ - atcacccacacgtcagcac - 3′
*Gabra1*	5′ - gcccactaaaattcggaagc - 3′	5′ - cttctgctacaaccactgaacg - 3′
*Gabra2*	5′ - acaaaaagaggatgggcttg - 3′	5′ - tcatgacggagcctttctct - 3′
*Gabra3*	5′ - cttgggaaggcaagaaggta - 3′	5′ - tggagctgctggtgttttct - 3′
*Gapdh*	5′ - agcttgtcatcaacgggaag - 3′	5′ - tttgatgttagtggggtctcg - 3′

### Statistical Analysis

For longitudinal results of body weights and fear conditioning, statistical analysis of mean values was conducted using two-way repeated measures ANOVA. Bonferroni’s post-hoc comparison was used to detect significant differences among groups. For non-longitudinal results of the elevated plus maze, memory test and gene expression, statistical analysis of mean values was conducted using one-way ANOVA. Fisher’s least significance difference (LSD) was used to detect significant differences between groups. Statistical significance was set at p<0.05. All analyses were conducted with the software SPSS 12.0 for Windows (SPSS, Chicago, III). Data are shown as means ± SEMs for all results.

## Results

Results are expressed in terms of the age of the mice, not the length of the experiment; i.e., “at 12 weeks” means “at the age of 12 weeks”.

### Effects of folate-methionine-choline-deficient (FMCD) diets on body weights

**Figure 1:**
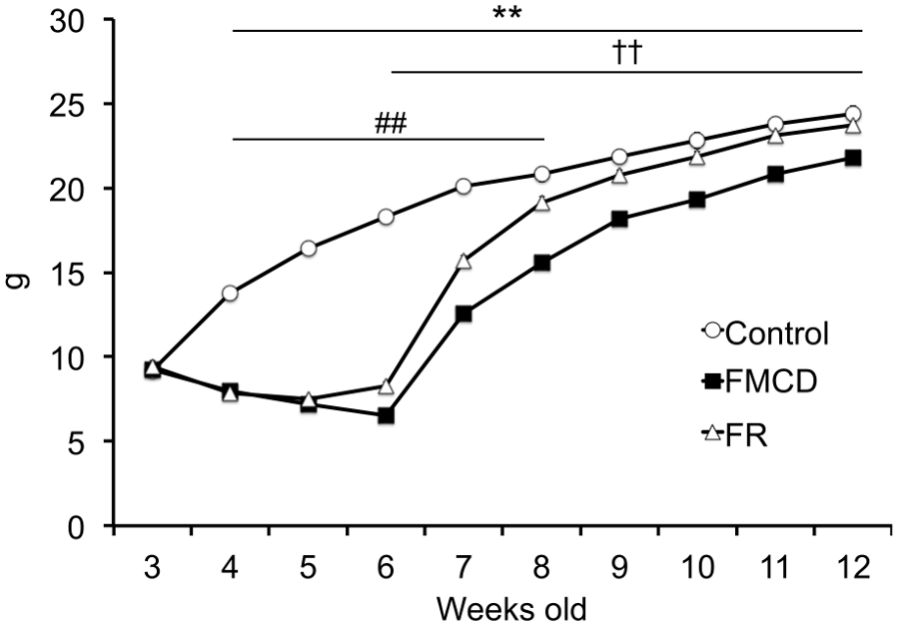
The effects of the folate-methionine-choline-deficient diets (FMCD) on body weight. The data represent the means ± SEMs (Control, n = 20; FMCD, n = 20; FR, n = 18). ** indicates *p*<0.01, Control vs. FMCD. ## indicates *p*<0.01, Control vs. FR. †† indicates *p*<0.01, FMCD vs. FR.

Two-way ANOVA with repeated measures showed a significant main effect of times (F(4.595, 252.715) = 910.128, *p*<0.001), group (F(2, 55) = 624.967) and a significant interaction between group and times (F(9.19, 252.715) = 44.529, *p*<0.001) (Fig. 1). Post hoc comparison indicated that the body weights of the FMCD group at 4–12 weeks of age were significantly reduced compared to the control group (p<0.001), and the body weights in the food restriction (FR) group at 4–8 weeks were significantly reduced compared to the control group (p<0.05). In addition, there were significant differences in body weights at 6 and 12 weeks between the FMCD group and FR group (p<0.001).

### Expression levels of *Dnmts* in the hippocampus

#### *Dnmt1*

**Figure 2:**
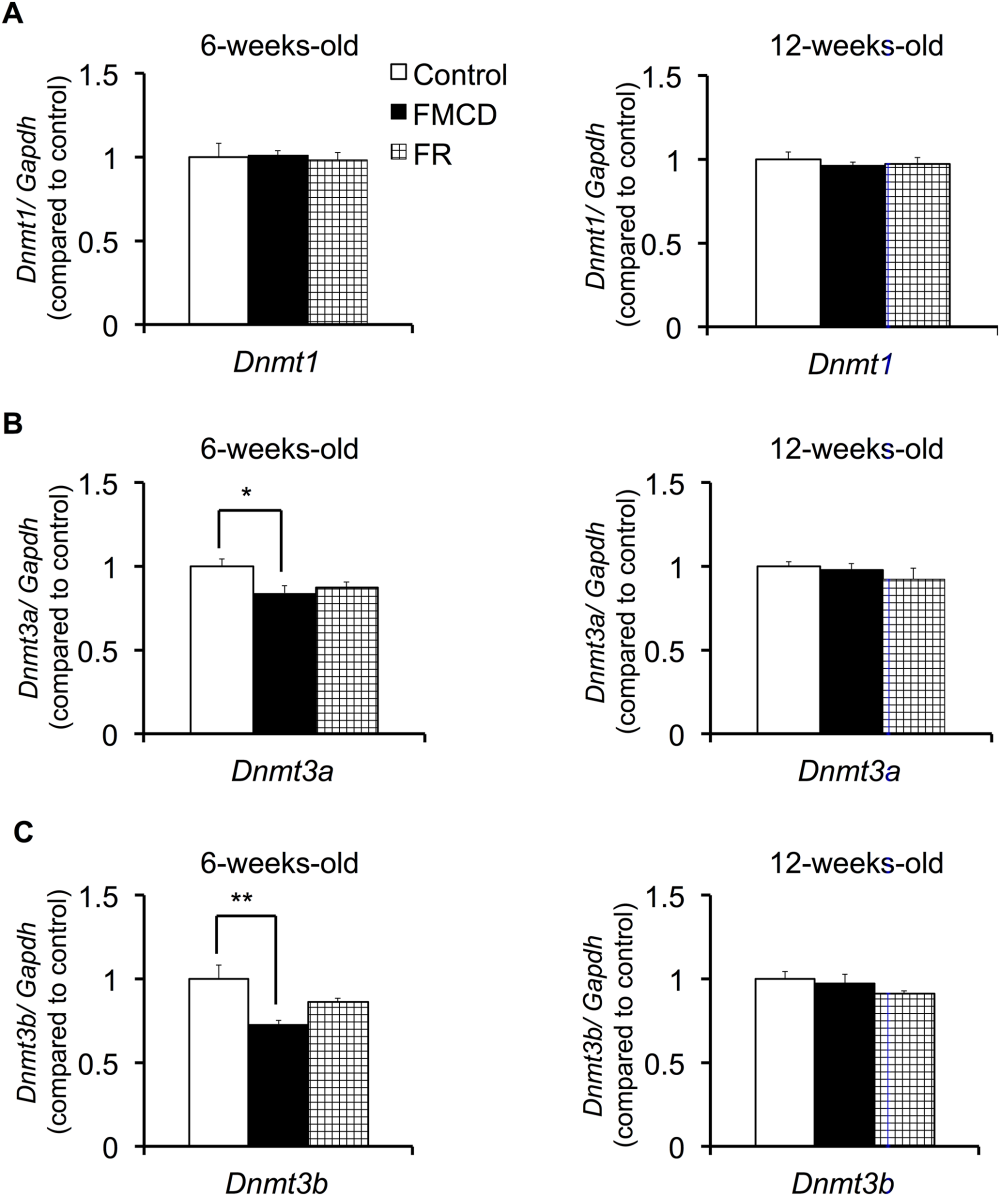
The effects of FMCD on the expression of DNA methyltransferases. Expressions of (A) *Dnmt1*, (B) *Dnmt3a and* (C) *Dnmt3b* in the dorsal hippocampus in juvenile (left) and adult mice (right). The data represent the means ± SEMs (Control, n = 5; FMCD, n = 5; FR, n = 5). * and ** indicate *p*<0.05 and *p*<0.01, respectively, Control vs. FMCD.

One-way ANOVA found no significant differences among groups in the expression of *Dnmt1*, either at 6 weeks (F(2, 12) = 0.071, p>0.05) or 12 weeks (F(2, 12) = 0.335, p>0.05) (Fig. 2A).

#### *Dnmt3a*

One-way ANOVA showed that at 6 weeks, *Dnmt3a* expressions significantly differed among groups (F(2, 12) = 4.034, p<0.05) (Fig. 2B). Fisher’s LSD showed that *Dnmt3a* expression was significantly lower in the FMCD group than in the control group (*p*<0.05). At 12 weeks *Dnmt3a* expressions no longer differed among groups (F(2, 12) = 0.722, p>0.05) (Fig. 2B).

#### *Dnmt3b*

At 6 weeks, *Dnmt3b* expressions significantly differed among groups as shown by one-way ANOVA (F(2, 12) = 6.758, p<0.05) (Fig. 2C). Fisher’s LSD showed that *Dnmt3b* expression was significantly lower in FMCD group than in the control group (*p*<0.01). At 12 weeks *Dnmt3b* expressions no longer differed significantly among groups (F(2, 12) = 1.29, p>0.05) (Fig. 2C).

### Effects of FMCD on contextual fear conditioning and memory test in juvenile mice

**Figure 3:**
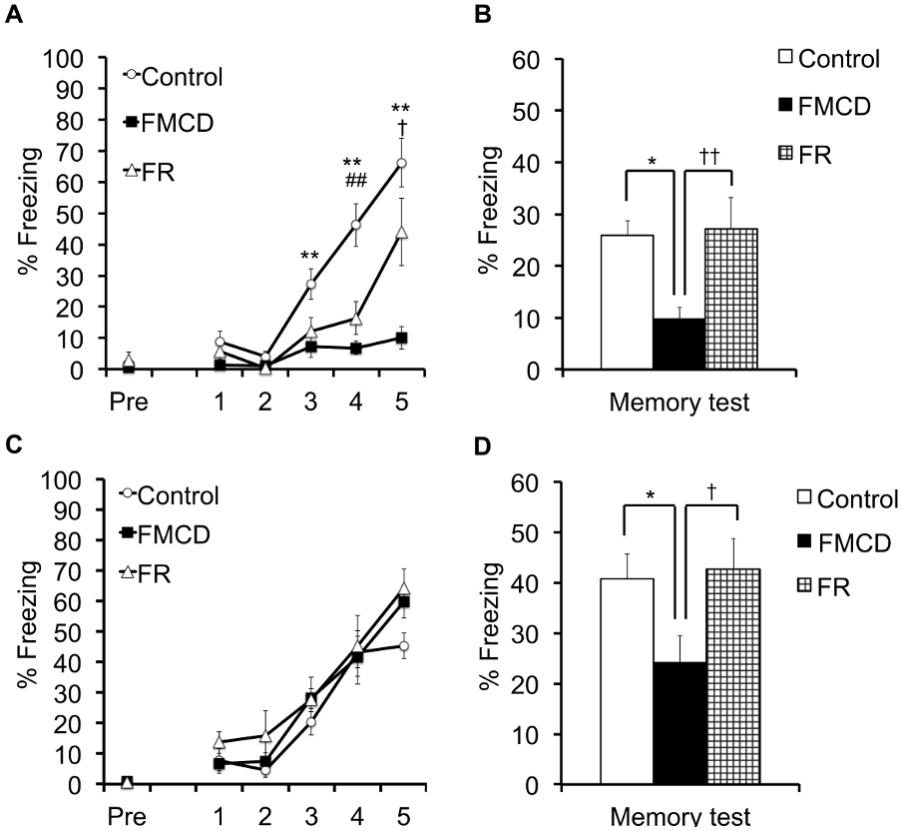
The effects of FMCD on contextual fear conditioning and memory test. (A) %Freezing during contextual fear conditioning in juvenile mice (Control, n = 10; FMCD, n = 10; FR, n = 10). (B) %Freezing during memory test in juvenile mice. (C) %Freezing during contextual fear conditioning in adult mice (Control, n = 9; FMCD, n = 10; FR, n = 10). (D) %Freezing during memory test in adult mice. The data represent the means ± SEMs. * and ** indicate *p*<0.05 and *p*<0.01, respectively, Control vs. FMCD. ## indicates *p*<0.01, Control vs. FR. † and †† indicate *p*<0.05 and p<0.01, respectively, FMCD vs. FR.

In the fear conditioning phase at 6 weeks, two-way ANOVA with repeated measures showed a significant main effect of the trials (F(2.482, 67.026) = 39.406, *p*<0.001) and group (F(2, 27) = 20.259, *p*<0.001) and a significant interaction between group and trials (F(4.965, 67.026) = 7.777, *p*<0.001). Post hoc comparison indicated that the %freezing at trials 3–5 was significantly lower in the FMCD group than in the control group (p<0.001) (Fig. 3A) and the %freezing at trial 4 was significantly lower in the FR group than in the control group (p<0.05) (Fig. 3A). In addition, there was a significant difference in the %freezing at trial 5 between the FMCD and FR groups (p<0.05) (Fig. 3A).

In the memory test phase at 6 weeks, the %freezing was significantly different among groups (F(2, 27) = 5.521, p<0.05) as shown by one-way ANOVA, and Fisher’s LSD showed a significant difference between the control and FMCD groups (p<0.05), with the FMCD group showing a lower %freezing (Fig. 3B). In addition, there was significant difference in %freezing at 6 weeks between the FMCD and FR groups (p<0.001), with the FMCD group showing a lower %freezing (Fig. 3B).

### Effects of FMCD on contextual fear conditioning and memory test in adult mice

In the fear conditioning phase at 12 weeks, two-way ANOVA with repeated measures showed that trials had a significant main effect (F(3.251, 84.516) = 54.709, *p*<0.001) whereas group did not show a significant main effect (F(2, 26) = 2.477, *p*>0.05); likewise, there was no significant interaction between group and trials (F(6.501, 84.516) = 0.614, *p*>0.05) (Fig. 3C). In the memory test phase at 12 weeks, the %freezing significantly differed among groups (F(2, 26) = 3.420, p<0.05) as shown by one-way ANOVA, and Fisher’s LSD showed a significant difference between the control and FMCD groups (p<0.05), with the FMCD group showing a lower %freezing (Fig. 3D). In addition, there was a significant difference in %freezing at 12 weeks between the FMCD and FR groups, with the FMCD group showing a lower %freezing (p<0.05) (Fig. 3D).

### Effects of the FMCD on elevated plus maze

**Figure 4:**
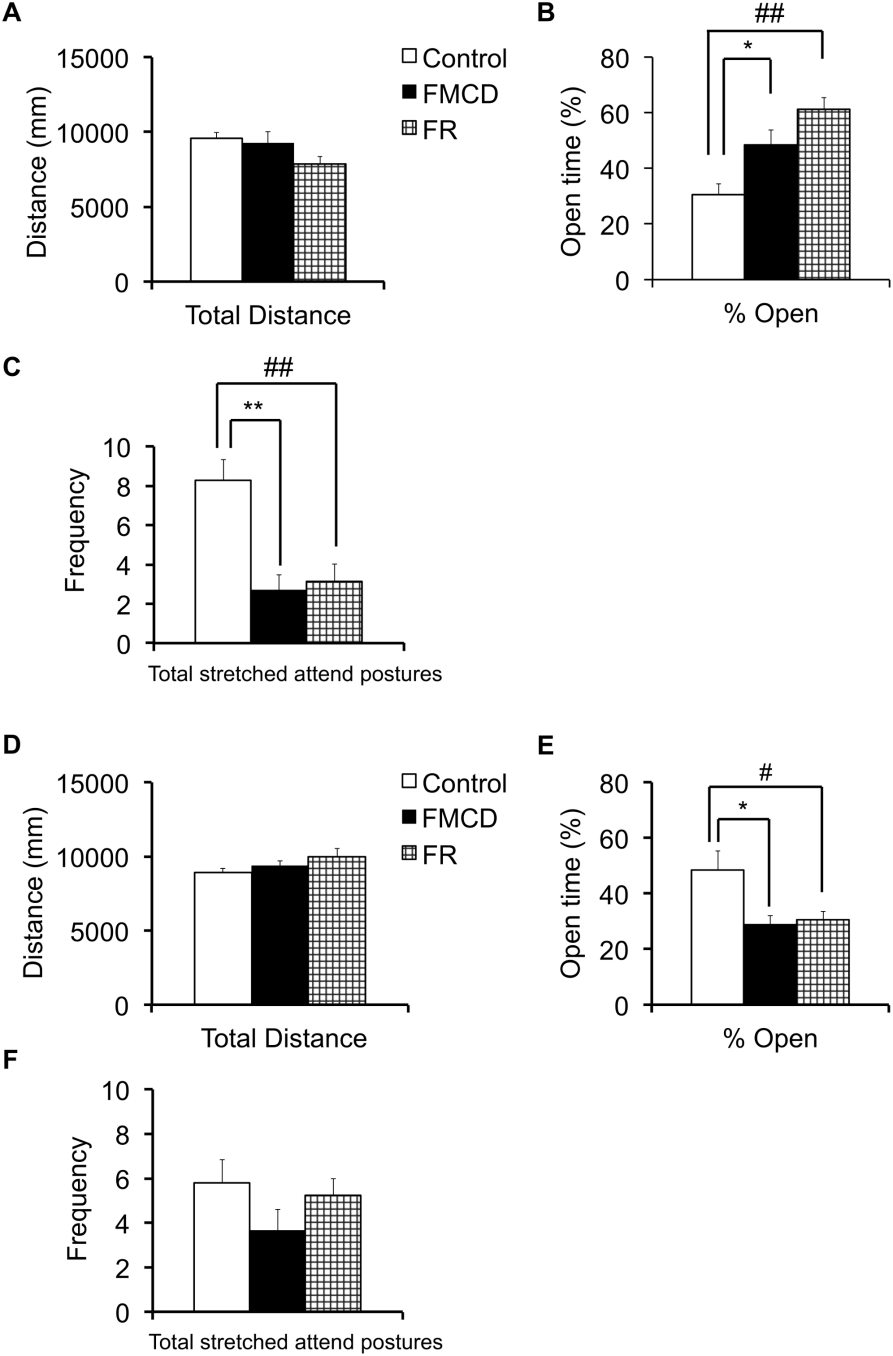
The effects of FMCD on locomotor activity and anxiety-like and risk-assessment behavior. (A) Locomotor activity during elevated plus maze in juvenile mice (Control, n = 10; FMCD, n = 9; FR, n = 8). (B) Anxiety-like behavior during elevated plus maze in juvenile mice. (C) Risk-assessment behavior during elevated plus maze in juvenile mice. (D) Locomotor activity during elevated plus maze in adult mice (Control, n = 9; FMCD, n = 8; FR, n = 8). (E) Anxiety-like behavior during elevated plus maze in adult mice. (F) Data on risk assessment behavior during elevated plus maze in adult mice. The data represent the means ± SEMs. * and ** indicate *p*<0.05 and *p*<0.01, respectively, Control vs. FMCD.

The results for the elevated plus maze are shown in Fig. 4. At 6 weeks, the total distance did not significantly differ among groups (F(2, 24) = 2.449, p>0.05) as shown by one-way ANOVA (Fig. 4A). However, one-way ANOVA revealed significant differences among groups in the %open arm at 6 weeks (F(2, 24) = 10.891, p<0.001), and Fisher’s LSD showed that the %open arm was significantly higher in both the FMCD and the FR group than in the control group (p<0.05 and p<0.001, respectively) (Fig. 4B). One-way ANOVA showed that the frequency of risk assessment at 6 weeks significantly differed among groups (F(2, 24) = 11.792, p<0.001), and Fisher’s LSD showed that the frequency of risk assessment was significantly lower in the FMCD group than in the control group (p<0.001) (Fig. 4C). The frequency of risk assessment was also significantly lower in the FR group than in the control group (p<0.01) (Fig. 4C).

At 12 weeks, one-way ANOVA found no significant differences among groups in the total distance (F(2, 22) = 1.923, p>0.05) (Fig. 4D), but did find significant differences among group in the %open arm at 12 weeks (F(2, 22) = 4.916, p<0.05) (Fig. 4E), Fisher’s LSD showed that the %open arm was significantly lower in the FMCD group than in the control group (p<0.05) (Fig. 4E). In addition, the %open arm at 12 weeks was significantly lower in the FR group than in the control group (p<0.05) (Fig. 4E). One-way ANOVA showed no significant differences among groups in the frequency of risk assessment at 12 weeks (F(2, 22) = 1.395, p>0.05) (Fig. 4F).

### Effects of the FMCD on gene expressions in juvenile mice

**Figure 5:**
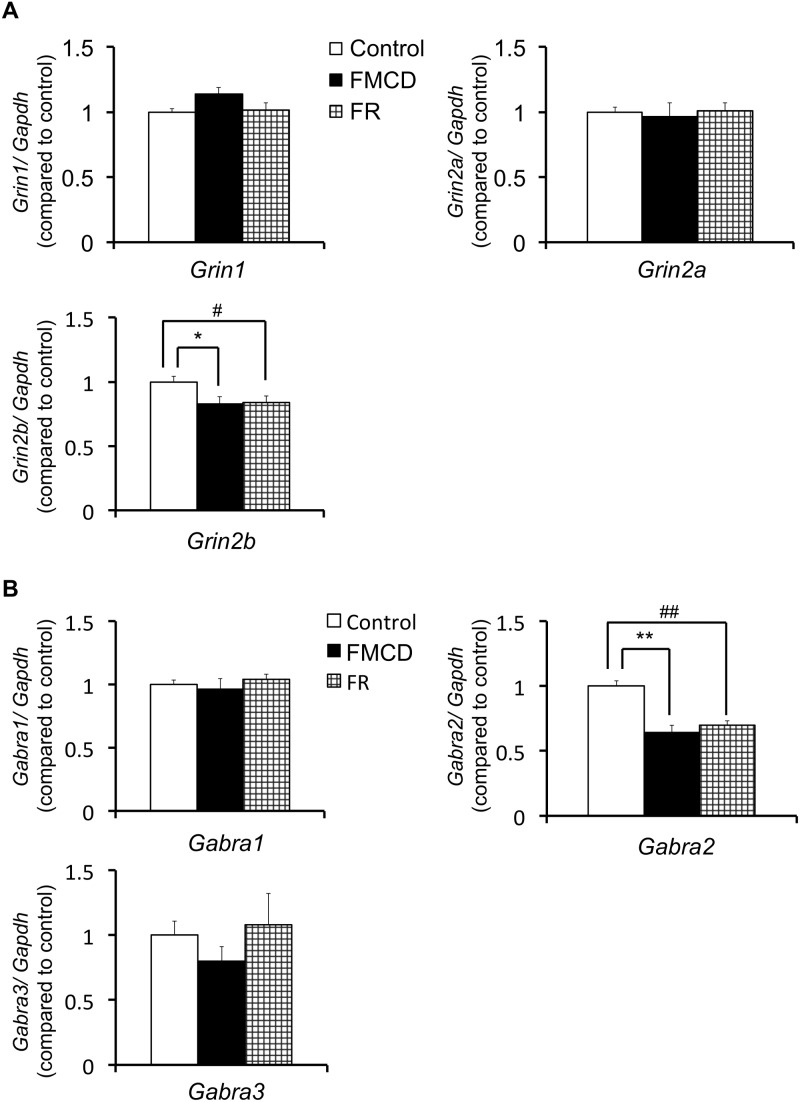
The effects of FMCD on gene expressions in the dorsal hippocampus in juvenile mice. (A) Gene expressions of *Grin1*, *Grin2a* and *Grin2b* (Control, n = 5; FMCD, n = 5; FR, n = 5). (B) Gene expressions of *Gabra1*, *Gabra2* and *Gabra3* (Control, n = 5; FMCD, n = 5; FR, n = 5). The data represent the means ± SEMs. * and ** indicate *p*<0.05 and *p*<0.01, respectively, Control vs. FMCD. # and ## indicate *p*<0.05 and *p*<0.01, respectively, Control vs. FR.

The results for hippocampal gene expressions in juvenile mice are shown in Table 2 and Figure 5. At 6 weeks, neither the *Grin1* expression (F(2, 12) = 2.598, p>0.05) nor *Grin2a* expression (F(2, 12) = 0.087, p>0.05) significantly differed among groups by one-way ANOVA. However, the *Grin2b* expression at 6 weeks did differ significantly among groups (F(2, 12) = 3.916, p<0.05), with Fisher’s LSD showing that the *Grin2b* expression was significantly lower in the FMCD group and in the FR than in the control group (*p*<0.05, respectively). The expression of *Gabra1* at 6 weeks was not significantly different among groups (F(2, 12) = 0.483, p>0.05) whereas the expression of *Gabra2* was (F(2, 12) = 17.761, p<0.001); Fisher’s LSD showed that the *Gabra2* expression was significantly lower in both the FMCD group (*p*<0.001) and the FR group (*p*<0.01) than in the control group. The expression of *Gabra3* at 6 weeks did not significantly differ among groups (F(2, 12) = 0.74, p>0.05) by one-way ANOVA.

**Table 2:** Effects of FMCD on gene expressions in the dorsal hippocampus.

	6 weeks old				12 weeks old			
	Fold change				Fold change			
Gene name	Control	FMCD	FR	F	Control	FMCD	FR	F
*Dnmt1*	1.00	1.01	0.98	F_2,12_ = 0.071	1.00	0.96	0.97	F_2,12_ = 0.335
*Dnmt3a*	1.00	0.84*	0.87	F_2,12_ = 4.034	1.00	0.98	0.92	F_2,12_ = 0.722
*Dnmt3b*	1.00	0.73**	0.86	F_2,12_ = 6.758	1.00	0.98	0.91	F_2,12_ = 1.290
*Grin1*	1.00	1.14	1.02	F_2,12_ = 2.598	1.00	1.02	0.99	F_2,12_ = 0.436
*Grin2a*	1.00	0.97	1.00	F_2,12_ = 0.087	1.00	1.01	0.93	F_2,12_ = 0.608
*Grin2b*	1.00	0.83*	0.84#	F_2,12_ = 3.916	1.00	1.02	0.96	F_2,12_ = 0.345
*Gabra1*	1.00	0.97	1.04	F_2,12_ = 0.483	1.00	0.95	0.93	F_2,12_ = 0.689
*Gabra2*	1.00	0.64**	0.70^##^	F_2,12_ = 17.761	1.00	0.90	0.96	F_2,12_ = 0.667
*Gabra3*	1.00	0.80	1.08	F_2,12_ = 0.740	1.00	0.66*	0.80	F_2,11_ = 4.310

*and **indicate *p*<0.05 and *p*<0.01, respectively, Control vs. FMCD.

# and ^##^indicate *p*<0.05 and *p*<0.01, respectively, Control vs. FR.

### Effects of the FMCD on gene expressions in adult mice

**Figure 6:**
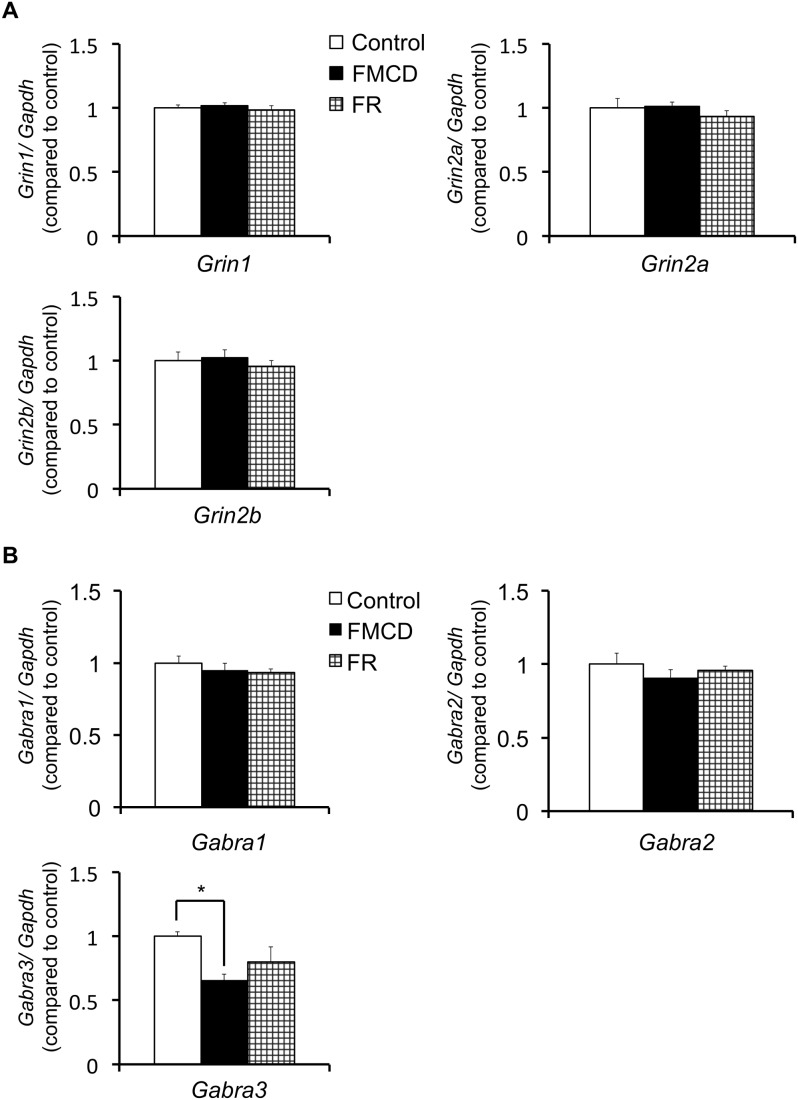
The effects of FMCD on gene expressions in the dorsal hippocampus in adult mice. (A) Gene expressions of *Grin1*, *Grin2a* and *Grin2b* (Control, n = 5; FMCD, n = 5; FR, n = 5). (B) Gene expressions of *Gabra1*, *Gabra2* and *Gabra3* (Control, n = 4–5; FMCD, n = 5; FR, n = 5). The data represent the means ± SEMs. * indicates *p*<0.05, Control vs. FMCD.

The results for gene expressions in the hippocampus are shown in Table 2 and Figure 6. At 12 weeks, neither the expression of *Grin1* (F(2, 12) = 0.436, p>0.05) nor the expressions of *Grin2a* (F(2, 12) = 0.608, p>0.05) or *Grin2b* (F(2, 12) = 0.345, p>0.05) were significantly different among groups, as shown by one-way ANOVA. At 12 weeks, the expressions of *Gabra1* (F(2, 12) = 0.689, p>0.05) and *Gabra2* (F(2, 12) = 0.667, p>0.05) did not significantly differ among groups, whereas the expression of *Gabra3* did (F(2, 11) = 4.31, p<0.05); Fisher’s LSD showed that the expression of *Gabra3* was significantly lower in the FMCD group than in the control group (*p*<0.05).

## Discussion

The major findings of our study were as follows. (1) Feeding the FMCD to mice from 3 to 6 weeks of age reduced gene expressions of *Dnmt3a* and *3b*, but these changes were reversed by subsequent feeding of a normal diet supplemented with methyl donors from 6 to 12 weeks of age. (2) Feeding the FMCD diet from 3 to 6 weeks totally impaired acquisition of hippocampus-dependent fear memory. Feeding the normal diet for the subsequent 6 weeks reversed the impaired acquisition of hippocampus-dependent memory; however, the consolidation or retrieval of the acquired fear memory was impaired. Additionally, restriction of food intake from 3 to 6 weeks induced the delayed acquisition of hippocampus-dependent fear memory, but this change was reversed by feeding of the normal diet ad libitum for the subsequent 6 weeks. (3) Feeding the mice the FMCD diet or restricting food intake from 3 to 6 weeks reduced anxiety-like behavior and the number of risk assessments, but in turn, anxiety-like behavior appeared by ad libitum intake of the normal diet after these experimental diets in both groups. (4) Feeding mice the FMCD diet or restricting food intake from 3 to 6 weeks of age induced low expression of *Grin2b* and *Gabara2* in the hippocampus, but these changes were reversed by ad libitum intake of the normal diet from 6 to 12 weeks. However, reduced gene expression of *Gabra3* was still observed in the FMCD group at 12 weeks of age.

The DNA methyltransferases are a family of enzymes that catalyze the transfer of a methyl group from S-adenosylmethionine (SAM) to the cytosine of a CpG dinucleotide. In mice, three DNA methyltransferases have been identified: *Dnmt1*, *Dnmt3a* and *Dnmt3b* [30], [31]. *Dnmt1* is the most abundant DNA methyltransferase in mammalian cells, which preferentially methylates hemi-methylated DNA [32]. Many studies have suggested that *Dnmt3a* and *Dnmt3b* are *de* *novo* DNA methyltransferases that methylate unmethylated CpG sites [31], [32], [33], [34]. By contrast, Chen et al. (2003) demonstrated that DNMT3a and DNMT3b have both *de* *novo* and maintenance functions *in* *vivo*, and are required for genome-wide *de* *novo* methylation [35]. Moreover, it has been reported that reduced expression of DNA methyltransferases induces global hypomethylation of tumors [36]. In addition, Liu et al. (2011) revealed that the downregulation of *Dnmt3a* and *Dnmt3b* in low-dose formaldehyde-treated cells inhibited *de* *novo* DNA methylation [37]. In our study, feeding mice a folate-methionine-choline-deficient diet from 3 to 6 weeks reduced gene expressions of *Dnmt3a* and *Dnmt3b*. Previous studies and our results suggest that the decreased expression of both *Dnmt3a* and *Dnmt3b* due to methyl donor deficiency during the developmental period might cause rearrangement of DNA methylation patterns in the brain. Furthermore, our results suggested that the decreased expressions of both *Dnmt3a* and *Dnmt3b* were reversed by feeding the diet supplemented with methyl donors after the deficiency period. Weaver et al. (2004) reported that altered DNA methylation was observed in the hippocampus of pups exposed to insufficient maternal behavior but that alteration was reversed by a specific drug such as histone deacetylase inhibitor [38]. Taken together, these findings indicate that the growth environment during the postnatal developmental period affects DNA methylation-associated factors and that the altered status of DNA methylation can be reversed by appropriate treatment thereafter.

Behavioral tests revealed that the fear response and anxiety behavior were affected in both the FMCD and food-restriction groups, but the alterations were different between the two groups. A marked difference appeared in the results of fear conditioning in juvenile mice (6 weeks of age) exposed to the two types of food restriction for 3 weeks. In the process of acquiring memory, new memories are stabilized after their acquisition, a process called consolidation. We observed that mice fed with the FMCD showed highly impaired acquisition of fear memory, which led to a very low level of freezing during the memory test the subsequent day. Restriction of food intake induced delayed acquisition of fear memory, but that delay did not affect the freezing level during the memory test, i.e., the consolidation ability of the acquired fear was intact (Fig. 3A). These results suggest that although complete deficiency of methyl donors impaired the acquisition of fear memory, low intake of methyl donors can prevent the impairment of acquisition, consolidation and retrieval of memory. Interestingly, up-regulation of hippocampal *Dnmt3a* and *3b* mRNAs was observed after fear conditioning in a previous rat study [11], and *Dnmt* inhibition blocked synaptic plasticity in a hippocampal slice [14]. Thus, our results support those previous results and suggest that *Dnmt* also plays an important role in acquisition of hippocampal memory in mice. It should be emphasized that a normal diet after 3-week restriction of methyl donors in the developmental period seemed to recover the fear-memory acquisition but that impaired memory was still observed in adults (12 weeks) (Fig. 3D). Lack of methyl donors in the developmental period might have caused persistent impairment of the capacity for consolidation or retrieval of acquired fear.

As to the results of elevated-plus maze test, both types of food restriction appeared to alter the anxiety-like behavior measured by the time spent in the open arm in both the juvenile (6 weeks) and adult (12 weeks) mice groups in opposite ways (Fig. 4). Both experimental groups showed decreased anxiety-like behavior in their juvenile period, but elevated anxiety-like behavior in adulthood, after ad libitum intake of a normal diet. There have been inconsistent results in previous studies with animals fed with diets lacking long-lasting methyl donors such as Vitamin Bs and choline after weaning. Although with a different time schedule, one such study with male rats reported a decrease in anxiety-like behavior, supporting our results [39]. In contrast, another report with male and female mice did not observe such decrease in anxiety-like behavior [40]. Alternatively, because behavioral changes in our study were observed in very similar ways in both experimental groups, it should be considered that the food restriction itself might have had the anxiolytic effect on juvenile mice, consistent with previous dietary restriction studies [41], [42], [43]. In the present study, locomotor activities among groups did not affect behavioral results because the total distance during the EPM and %freezing during acclimation period were not significantly different among groups.

In the current study, we investigated the hippocampal mRNA expressions of NMDA receptor subunits of NR1, NR2A and NR2B, and GABA receptor subunits of a2 and a3 to determine whether expressions of those receptors were involved in the mechanism behind the behavioral changes in the experimental mice groups. The NMDA receptor is considered to play a crucial role in the formation of hippocampal memory. In our study, FMCD and FR mice revealed low expression of the NR2B gene, suggesting that the reduced intake of methyl donors may induce the decrease of NR2B gene expression by global rearrangement of DNA methylation patterns. Supporting this hypothesis, the expression of the NR2 gene is regulated by DNA methylation in the 5′-regulatory area [44]. Furthermore, memory acquisition and consolidation depends on the reactivation of the NMDA receptor in the hippocampus, and NR2B is critical in memory formation [45], [46], [47]. These studies and our results suggest that the stable expression of the NR2B gene is necessary for acquiring fear memory. Thus, the lack of fear acquisition in the FMCD group (Fig. 3A) can be explained on the basis of the alternation in the expression of the NR2B subunit as well. Importantly, mice fed a diet supplemented with essential one-carbon nutrients after exposure to FMCD reversed their expression of the NR2B gene. Taking folate, methionine and choline might have normalized the alteration of one-carbon metabolism caused by exposure to FMCD.

In addition to the reduced expression in the NR2B subunit gene, we found that the mRNA expression of *Gabra2* was reduced in both experimental groups at 6 weeks. It is known that activating GABA signaling is crucial to the development of NMDA receptor-mediated neural activity during the maturation of the brain [48]. The combination of decreased expressions of NMDA and GABA receptor genes might also have contributed to the imbalance between the excitatory/inhibitory neurotransmission via glutamate/GABA, which could lead to the alteration of reduced anxiety-like behaviors as well as the reduced fear acquisition in the experimental groups at 6 weeks. In addition, it is important to note that GABA transmission is initially excitatory but becomes inhibitory during the early postnatal period in rodents [49], [50], and such maturation of GABAergic transmission is known to develop with increasing expression of chloride transporter KCC2 [51]. Because the food restriction in our experiments started at postnatal day 21 (3 weeks), when the rodent hippocampus is still in the developmental stage [52], [53], intracellular chloride homeostasis in the hippocampus of our experimental mice groups might have been altered due to the poor nutrition. Interestingly, ad libitum intake of a normal diet reversed the decreased expression of *Gabra2* but also induced an increase in anxiety-like behavior (Fig. 4 and 6); in addition, persistently reduced hippocampal expression of *Gabra3* was observed in the FMCD group. The expression of *Gabra3* in the hippocampus is relatively high compared to other brain regions [54], [55], and the alterations in GABA_A_ receptor subunit composition during the developmental period might influence later responses to stressors [56] and adult neurogenesis, which could serve as a fundamental substrate of anxiety that appears in adulthood [57].

Several limitations to the study should be noted. First, body weights were reduced by feeding the FMCD as shown in Figure 1. Rizki et al. (2006) revealed that feeding a methionine-choline-deficient diet induced the loss of body weight and the gain of energy consumption [29]. This observed weight loss might be due to lack of methionine, since a choline-folic acid-deficient diet with low methionine (0.18%) in a previous study did not induce weight loss [27]. Second, food satiation is known to affect anxiety-like behavior [42]. In our study, the FMCD group might have had food satiation, because this group had ad libitum access to the FCMD diet. In contrast, the FR group might not have had food satiation, because the FR group was fed a restricted amount of the normal diet. Thus, despite the anxiety-like behaviors being similar in the two experimental groups, some qualitative difference may have existed. Considering the two limitations described, we are planning further experiments with a choline-folic acid-deficient/low-methionine diet in the next study, which would attenuate the possible effect of the weight loss. Third, although alterations of several mRNA expressions including *Dnmt* strongly suggest that rearrangement of DNA methylation occurred in the brains of FMCD mice, our study does not include direct evidence. Further study showing the actual occurrence of the epigenetic alteration would be desirable. However, it should be noted that a previous study revealed that long-term reduction of dietary methyl donors induced the rearrangement of DNA methylation in the rat brain [27].

In summary, we examined the effects of FMCD exposure during the developmental period on emotional behaviors and hippocampal gene expressions in juvenile and adult mice. Feeding mice an FMCD diet during the developmental period induced severe impairment of memory acquisition and a decrease in anxiety-like behavior. Feeding the same mice a normal diet after the FMCD treatment partially reversed the behavioral alteration, but the adult mice still had impairment in fear-memory consolidation or retrieval while anxiety-like behavior was elevated. Gene expressions of *Dnmt3a* and *3b* in the hippocampus were decreased by the FMCD exposure, which strongly suggests the occurrence of DNA methylation rearrangement in the FMCD mice group. Changes in gene expressions of *Grin2b* and *Gabra2/3* might have been involved in the mechanism behind the behavioral alterations. Our study suggests that altering the one-carbon metabolic pathway in the developmental brain could affect emotional behavior and memory formation.
